# Serum phoenixin-14 levels in chronic kidney disease and hemodialysis patients: a cross-sectional study

**DOI:** 10.3389/fmed.2026.1896542

**Published:** 2026-09-07

**Authors:** Erdoğan Özdemir, Fatih Genç, Turgay Yılmaz, Deccane Düzenci

**Affiliations:** 1 Department of Internal Medicine, Elazığ Fethi Sekin City Hospital, Elazığ, Türkiye; 2 Department of Nephrology, Elazığ Fethi Sekin City Hospital, Elazığ, Türkiye; 3 Department of Intensive Care Unit, Elazığ Fethi Sekin City Hospital, Elazığ, Türkiye

**Keywords:** biomarker, chronic kidney disease, hemodialysis, phoenixin-14, renal function

## Abstract

**Background:**

Phoenixin-14 (PNX-14) is a bioactive peptide associated with mitochondrial function, oxidative stress responses, and cellular protection. Although experimental data suggest a possible role in renal injury, its serum levels in chronic kidney disease (CKD) have not been previously studied. Given the limitations of conventional renal biomarkers, PNX-14 may offer additional information regarding renal dysfunction in CKD.

**Methods:**

This single-center, prospective, observational, cross-sectional study included 140 adult patients with Stage 3 CKD (*n* = 30), Stage 4 CKD (*n* = 30), Stage 5 CKD (*n* = 30), and end-stage kidney disease receiving maintenance hemodialysis (HD) (*n* = 50). Serum PNX-14 was measured using a commercial ELISA kit, before and after dialysis in HD patients. Associations between PNX-14 and clinical and laboratory parameters were assessed using Spearman correlation. Receiver operating characteristic (ROC) curve analysis evaluated the discriminative performance of PNX-14 across CKD stages.

**Results:**

Serum PNX-14 levels increased progressively with advancing CKD stage, from a median of 17.88 (IQR 15.16–20.20) pg/mL in Stage 3–43.14 (IQR 23.78–95.71) pg/mL in the HD group (*p* < 0.001). *Post hoc* analysis showed that PNX-14 levels were significantly lower in Stage 3 than in Stage 5 and HD, in Stage 4 than in HD, and in Stage 5 than in HD. PNX-14 showed significant positive correlations with creatinine and parathyroid hormone (PTH) and a significant negative correlation with eGFR (all *p* < 0.001). In the pre-dialysis subgroup, PNX-14 remained significantly correlated with creatinine, eGFR, urea, and PTH. In HD patients, no significant difference was observed between pre-dialysis and post-dialysis PNX-14 levels (*p* = 0.152), and no significant correlation was found between PNX-14 and Kt/V. ROC analysis demonstrated good discriminative performance for distinguishing Stage 3 from advanced disease (Stage 5 + HD), with an AUC of 0.839 (95% CI: 0.755–0.913). The optimal cut-off was 19.59 pg/mL, with 87.5% sensitivity and 73.3% specificity.

**Conclusions:**

Serum PNX-14 levels increase with worsening CKD stage and are strongly associated with renal function parameters. Its limited change after HD and its discriminative performance suggest that PNX-14 may provide clinically relevant information regarding disease severity in CKD. Further prospective and longitudinal studies are needed to clarify its potential clinical utility.

## 1 Introduction

Chronic kidney disease (CKD) is a growing public health problem that affects a substantial proportion of the global population. According to the Global Burden of Disease 2023 study, the number of individuals aged 20 years and older living with CKD increased from 378 million in 1990 to 788 million in 2023, and CKD ranked as the ninth leading cause of death worldwide in the same year (1). The age-standardized prevalence of CKD is estimated to be approximately 14.2%, and the burden of the disease is greater in low- and middle-income countries where access to healthcare is limited. If current trends continue, mortality attributable to CKD is expected to double by 2040, highlighting the need to develop strategies for early diagnosis and monitoring (1, 2).

CKD is characterized by the progressive loss of kidney function and predisposes patients to serious complications such as cardiovascular disease, anemia, mineral and bone disorders, metabolic acidosis, and end-stage kidney disease (ESKD) (3, 4). Accurate staging of the disease and early prediction of its progression are therefore of great importance for the effective management of these complications. The 2024 KDIGO clinical practice guideline recommends estimated glomerular filtration rate (eGFR) and albuminuria as the principal parameters for the diagnosis and staging of CKD (3). However, these conventional markers have important limitations. Serum creatinine may be influenced by non-renal factors such as muscle mass, age, and nutritional status, and may remain within the normal range despite substantial nephron loss (5). Albuminuria, on the other hand, shows daily variability and may be affected by conditions such as hydration status, physical activity, and infection. Moreover, since kidney damage may develop in the absence of albuminuria, particularly in early-stage or non-proteinuric conditions, albuminuria may not adequately reflect the true severity of the disease. While it primarily reflects glomerular injury, it represents processes such as tubulointerstitial damage, inflammation, and fibrosis only to a limited extent (6). For these reasons, there remains a need for novel biomarkers that better reflect the pathophysiological processes of CKD and are more reliable for clinical use.

Current biomarker research in CKD has focused on candidate markers, particularly NGAL, KIM-1, and CKD273, with these studies being conducted predominantly in diabetic nephropathy and other types of CKD (5–7). Although these biomarkers have shown promising diagnostic and prognostic potential, they have not yet entered routine clinical practice because of limited specificity, high cost, and difficulties related to standardization and clinical validation (5, 6). This situation indicates that the need for biologically meaningful and clinically applicable novel biomarkers still persists.

The phoenixin peptide family consists of two main isoforms, PNX-14 and PNX-20, which are derived through proteolytic processing from the precursor protein SMIM20 (small integral membrane protein 20) (8, 9). Phoenixin-14 (PNX-14) is a 14-amino-acid peptide and is the most extensively studied isoform. Acting through GPR173 (G protein-coupled receptor 173), PNX-14 was initially identified as a regulator of the reproductive axis; however, subsequent studies have demonstrated that it also exerts cardioprotective, neuroprotective, and anti-inflammatory effects (9–12). In addition, SMIM20, the precursor protein of PNX-14, has been reported to play a role in the formation of mitochondrial complex IV (cytochrome c oxidase), which is essential for cellular energy production (11). These findings suggest that PNX-14 may have a biologically meaningful role in conditions in which mitochondrial dysfunction is a prominent feature.

With regard to the role of PNX-14 in kidney diseases, evidence from the literature is limited but suggestive. In experimental rat studies, administration of PNX-14 in an ischemia/reperfusion-induced acute kidney injury model was shown to reduce serum BUN and creatinine levels, increase the activity of antioxidant enzymes such as superoxide dismutase (SOD) and catalase (CAT) as well as glutathione levels, decrease malondialdehyde levels, and ameliorate histopathological kidney damage (13). Considering the major pathophysiological processes of CKD, including accumulation of uremic toxins, chronic inflammation, oxidative stress, and metabolic acidosis, a possible relationship between CKD progression and PNX-14 physiology may be hypothesized. For all these reasons, the aim of the present study was to compare serum PNX-14 levels across different stages of chronic kidney disease and in patients undergoing hemodialysis (HD), to evaluate the effect of HD on PNX-14 kinetics, and to further examine the correlations of PNX-14 with clinical and laboratory parameters as well as its diagnostic value.

## 2 Materials and methods

### 2.1 Study design and ethical approval

This study was a single-center, prospective, observational, cross-sectional study conducted in May 2026. The study protocol was approved by the Ethics Committee of Elazığ Fethi Sekin City Hospital (decision no: 2026/29-04, decision date: 07.05.2026), and all procedures were carried out in accordance with the principles of the Declaration of Helsinki. Written informed consent was obtained from all participants.

### 2.2 Study population

Patients aged 18 years and older who were being followed in the nephrology outpatient clinic and HD unit were included in the study. The diagnosis and staging of CKD were established according to the KDIGO 2024 Clinical Practice Guideline (3). Accordingly, patients with Stage 3 CKD (*n* = 30), Stage 4 CKD (*n* = 30), Stage 5 CKD (*n* = 30), and patients receiving HD for ESKD (*n* = 50) were included in the study. Patients were enrolled consecutively according to predefined inclusion and exclusion criteria. Consecutive eligible patients attending the nephrology outpatient clinic and the HD unit during the study period were screened, and all who met the criteria and provided consent were enrolled, with no further selection. Baseline demographic and laboratory data were retrieved from the hospital information system on the day of sampling. Concomitant medication use was reviewed at screening only in order to apply the exclusion criteria, in particular the exclusion of patients receiving immunosuppressive therapy. Beyond this, comorbidity status and drug treatment were not recorded as part of the approved study protocol and could not be retrieved retrospectively, so they were not available for analysis; this is acknowledged as a limitation.

#### 2.2.1 Inclusion criteria

age 18 years or older; diagnosis of Stage 3, 4, or 5 CKD according to KDIGO 2024, or enrollment in a maintenance HD program; and at least 3 months of clinical stability, defined as the absence of acute clinical deterioration related to kidney disease or hospitalization during this period.

#### 2.2.2 Exclusion criteria

age younger than 18 years; active infection or sepsis; active malignancy; pregnancy; liver cirrhosis or severe hepatic failure; autoimmune disease; history of renal transplantation; acute kidney injury; and use of immunosuppressive medications.

### 2.3 Patient groups and CKD staging

Patients were classified into four groups according to the KDIGO 2024 criteria. The race-free CKD-EPI 2021 equation was used to calculate eGFR (14). The groups were defined as follows: Stage 3 CKD (eGFR 30–59 mL/min/1.73 m^2^), Stage 4 CKD (eGFR 15–29 mL/min/1.73 m^2^), Stage 5 CKD (eGFR <15 mL/min/1.73 m^2^), and the HD group (patients receiving maintenance HD treatment).

### 2.4 Hemodialysis protocol

All HD patients underwent dialysis sessions three times per week, with each session lasting four hours. Both low-flux and high-flux dialysers were in routine use in the unit; however, the membrane type applied in individual sessions was not recorded at the patient level, and dialysis was performed with standard vascular access via an arteriovenous fistula. Dialysis parameters were set in accordance with international KDOQI/KDIGO guidelines, with a blood flow rate of 300–320 mL/min, a dialysate flow rate of 500 mL/min, and a target Kt/V of ≥1.4. A standard bicarbonate-based dialysate was used (bicarbonate 35 mmol/L, calcium 1.25–1.5 mmol/L). Kt/V values for all patients were obtained from routine clinical records and were included in the correlation and subgroup analyses.

### 2.5 Collection of routine blood and urine samples

Venous blood samples were collected from patients with Stage 3, 4, and 5 CKD in the morning after an 8-hour fasting period. In the HD group, two serum samples were obtained: one before the dialysis session (pre-dialysis) and one immediately after completion of the session (post-dialysis). Pre-dialysis samples were drawn from the arterial needle immediately after cannulation and before the initiation of blood flow or the administration of heparin, at the midweek session. Post-dialysis samples were obtained at the end of the fourth hour, immediately before disconnection, after the blood pump had been slowed to 50–100 mL/min for at least 15 s in order to minimize access recirculation, in accordance with the slow-flow technique recommended for post-dialysis urea sampling. Samples were not corrected for hemoconcentration arising from ultrafiltration, since ultrafiltration volumes were not recorded; this is addressed in the limitations. For the albumin-to-creatinine ratio (ACR), spot urine samples were collected from the first morning urine of patients with Stage 3, 4, and 5 CKD. All samples were analyzed on the same day in the same laboratory.

### 2.6 Measurement of PNX-14

Serum PNX-14 levels were measured using the sandwich ELISA method with a commercially available human PNX-14 ELISA kit (Phoenixin 14 ELISA Kit, Catalog No: E7050Hu; BT-Lab, Bioassay Technology Laboratory, Shanghai, China), in accordance with the manufacturer's instructions. The assay had a measurement range of 2.5–160 pg/mL and an analytical sensitivity of 1.22 pg/mL, with intra-assay and inter-assay coefficients of variation of <8% and <10%, respectively. Samples exceeding the upper limit of detection were reanalyzed after appropriate dilution. Absorbance measurements were performed using a BIO-TEK ELX800 microplate reader and a BIO-TEK ELX50 washer. Results were expressed in pg/mL.

### 2.7 Laboratory and clinical parameters

In all patients, serum creatinine, urea, albumin, phosphorus, calcium, uric acid, parathyroid hormone (PTH) and hemoglobin were measured, and eGFR was calculated. Serum creatinine, urea, albumin, phosphorus, calcium and uric acid were determined on an automated clinical chemistry analyzer (AU5800; Beckman Coulter Inc., Miami, FL, USA) using the manufacturer's proprietary reagents; creatinine was measured by the Jaffe method and albumin by the bromocresol green method. Intact PTH was measured on a DxI 800 immunoassay system (Beckman Coulter Inc., Miami, FL, USA). Hemoglobin was measured on a DxH 800 hematology analyzer (Beckman Coulter Inc., Miami, FL, USA). Urine albumin was measured by an immunoturbidimetric method and urine creatinine on the same AU5800 platform; the spot urine albumin-to-creatinine ratio (ACR) was reported in mg/g. Bicarbonate (HCO₃⁻) and pH were measured on an ABL90 FLEX blood gas analyzer (Radiometer Medical ApS, Brønshøj, Denmark). Body mass index (BMI) was calculated as kg/m^2^. All routine assays were subject to daily internal quality control using the manufacturer's control materials and to external quality assessment through the Randox International Quality Assessment Scheme (RIQAS) and the KBUDEK external quality assessment program of the Turkish Society of Clinical Biochemistry Specialists.

### 2.8 Statistical analysis

All statistical analyses were performed using IBM SPSS Statistics v29 (IBM Corp., Armonk, NY, USA). Continuous variables were expressed as mean ± standard deviation (SD) or median (interquartile range, IQR), according to their distribution, while categorical variables were presented as number and percentage. The normality of data distribution was assessed for each continuous variable within each study group using the Shapiro–Wilk test, and homogeneity of variances was assessed using Levene's test. Variables that were normally distributed in all groups with homogeneous variances were summarized as mean ± SD and compared across groups using one-way analysis of variance (ANOVA), whereas variables that departed from normality in at least one group were summarized as median (IQR) and compared using the Kruskal–Wallis test. Accordingly, non-parametric methods were used for PNX-14 for all primary comparisons and correlations. For pairwise comparisons of PNX-14 between groups following a significant Kruskal–Wallis test, the Mann–Whitney U test was applied, and the Holm-Bonferroni sequential correction method was used to adjust for multiple comparisons (family-wise error rate *α*=0.05). The Holm-Bonferroni procedure was applied across the six pairwise stage comparisons as a single family of tests. Dunn's test with Bonferroni adjustment was additionally performed as a sensitivity analysis. Categorical variables were compared using the chi-square test. Serum PNX-14 was compared between men and women using the Mann–Whitney U test in the whole cohort, in the predialysis and HD groups and within each CKD stage, and the group-by-sex interaction was assessed by two-way analysis of variance on log-transformed PNX-14. The Wilcoxon signed-rank test was used to compare pre-dialysis and post-dialysis PNX-14 levels. The relationships between PNX-14 levels and clinical parameters were evaluated using Spearman correlation analysis. The diagnostic performance of PNX-14 was assessed by receiver operating characteristic (ROC) curve analysis. Optimal cut-off values were determined using the Youden index (maximum sensitivity + specificity - 1). The area under the ROC curve (AUC) and 95% confidence intervals were calculated using the bootstrap method (1000 iterations). Additional statistical analyses, including ordinal logistic regression, Spearman rank correlation, and effect size (Cohen's d) calculations, were also performed (Supplementary Tables S1–S3). To determine whether serum PNX-14 was independently associated with renal function, multivariable linear regression was performed with natural log-transformed PNX-14 as the dependent variable. Models were adjusted for eGFR, age, sex, BMI and PTH, and multicollinearity was assessed using variance inflation factors (all < 3). A parallel model was fitted in the pre-dialysis subgroup with the addition of the spot urine albumin-to-creatinine ratio. Multivariable binary logistic regression adjusted for age, sex and BMI was used to assess the independent association of PNX-14 with advanced disease. No *a priori* sample size calculation was performed; group sizes were determined by the number of eligible patients available during the enrolment period. A *post hoc* power analysis was therefore conducted for the primary comparison. In all analyses, a *p* value of <0.05 was considered statistically significant.

## 3 Results

### 3.1 Characteristics of the study population

A total of 140 patients with CKD were included in the study [Stage 3 (*n* = 30), Stage 4 (*n* = 30), Stage 5 (*n* = 30), and HD (*n* = 50)]. The median age of the patients was 64.0 (IQR 52.0–73.0) years, and 62.1% were male. Statistically significant differences were observed among the groups in terms of age and BMI (both *p* < 0.001) and sex distribution (*p* = 0.001). Kidney function parameters (creatinine, eGFR) and hemoglobin levels also differed significantly between the groups (*p* < 0.001). Detailed demographic and clinical characteristics of the study population are presented in Table 1.

**Table 1:** Demographic and clinical characteristics of the study population.

Parameter	Stage 3 (*n* = 30)	Stage 4 (*n* = 30)	Stage 5 (*n* = 30)	HD (*n* = 50)	*P* value
Age (years)	71.0 (64.3–75.0)	64.5 (58.5–74.8)	68.5 (61.0–75.0)	54.0 (38.0–68.0)	<0.001
Male sex, *n* (%)	25 (83.3)	16 (53.3)	11 (36.7)	35 (70.0)	0.001
BMI (kg/m^2^)	30.5 (28.4–33.1)	27.7 (25.4–32.2)	27.4 (25.0–30.4)	24.4 (21.3–27.8)	<0.001
Creatinine (mg/dL)	1.42 (1.28–1.62)	2.43 (1.92–2.72)	4.31 (3.85–5.19)	8.44 (7.18–10.70)	<0.001
eGFR (mL/min/1.73 m^2^)	46.0 (39.0–58.8)	27.5 (20.5–29.0)	11.0 (8.5–14.0)	6.0 (5.0–7.0)	<0.001
Hemoglobin (g/dL)	13.8 ± 1.6	12.5 ± 1.7	11.1 ± 1.5	11.5 ± 1.4	<0.001
Serum Albumin (g/dL)	4.00 (3.80–4.30)	3.95 (3.73–4.28)	3.80 (3.45–4.05)	3.90 (3.63–4.07)	0.023
Spot Urine ACR (mg/g)	310 (144–488)	902 (651–1334)	2087 (1505–4434)	—	<0.001

Data are presented as mean ± SD for hemoglobin, as median (interquartile range, Q1–Q3) for all other continuous variables, and as *n* (%) for categorical variables. Normality was assessed within each group using the Shapiro–Wilk test. Hemoglobin was the only continuous variable normally distributed in all four groups with homogeneous variances (Levene's test, *p* = 0.728) and was compared using one-way ANOVA; all other continuous variables were compared using the Kruskal–Wallis test, and categorical variables using the chi-square test. Spot urine ACR was available only for pre-dialysis patients (CKD stages 3–5) and was therefore compared across these three groups only. BMI, body mass index; eGFR, estimated glomerular filtration rate; ACR: albumin-to-creatinine ratio; HD, hemodialysis.

### 3.2 Serum PNX-14 levels and CKD stages

Serum PNX-14 levels increased progressively with advancing CKD stage (Table 2). PNX-14 levels [median (IQR)] were 17.88 (15.16–20.20) pg/mL in Stage 3, 21.95 (16.74–29.17) pg/mL in Stage 4, 23.65 (20.45–39.63) pg/mL in Stage 5, and 43.14 (23.78–95.71) pg/mL in the HD group. The difference among the groups was statistically significant (Kruskal–Wallis H = 40.006, *p* < 0.001). The corresponding means ± SD are presented in Table 2.

**Table 2:** Serum PNX-14 levels according to CKD stages.

Group	n	Mean ± SD	Median	IQR (Q1–Q3)	Min-Max
Stage 3	30	19.37 ± 11.16	17.88	15.16–20.20	1.68–58.72
Stage 4	30	26.84 ± 19.08	21.95	16.74–29.17	7.94–97.03
Stage 5	30	42.50 ± 45.97	23.65	20.45–39.63	2.64–227.39
HD	50	67.02 ± 53.63	43.14	23.78–95.71	9.54–196.30

Data are presented as pg/mL. SD, standard deviation; IQR, interquartile range reported as Q1–Q3; HD, hemodialysis. Between-group comparison: Kruskal–Wallis test (H = 40.006, *p* < 0.001). Because serum PNX-14 was non-normally distributed in all groups (Shapiro–Wilk *p* < 0.001), the median (IQR) is the primary summary measure; means ± SD are shown for descriptive completeness.

In the *post hoc* analyses performed with Holm-Bonferroni correction (Table 3), PNX-14 levels were found to be significantly lower in Stage 3 than in Stage 5 (*p* < 0.001) and the HD group (*p* < 0.001), in Stage 4 than in the HD group (*p* < 0.001), and in Stage 5 than in the HD group (*p* = 0.017).

**Table 3:** Pairwise comparisons of Serum PNX-14 levels between CKD stages.

Comparison	*P* value	Adjusted *α*	Significance
Stage 3 vs. HD	<0.001	0.0083	***
Stage 4 vs. HD	<0.001	0.0100	***
Stage 3 vs. Stage 5	<0.001	0.0125	***
Stage 5 vs. HD	0.017	0.0167	***
Stage 3 vs. Stage 4	0.044	0.0250	NS
Stage 4 vs. Stage 5	0.071	0.0500	NS

Pairwise comparisons using Mann–Whitney U test with Holm-Bonferroni correction (α=0.05). Comparisons ordered by *p*-value. HD, hemodialysis; NS, not significant.

*** *p* < adjusted α (statistically significant).

The stage-dependent increase in PNX-14 was further supported by complementary analyses (Supplementary Tables S1–S3). When CKD stage was modeled as an ordinal outcome, each 10 pg/mL increase in serum PNX-14 was associated with higher odds of more advanced disease in the unadjusted model (OR = 1.295, 95% CI: 1.158–1.448, *p* < 0.001) and after adjustment for age, sex, and BMI (OR = 1.247, 95% CI: 1.122–1.385, *p* < 0.001); this association was attenuated and no longer significant after additional adjustment for creatinine and eGFR (OR = 1.022, 95% CI: 0.878–1.189), consistent with the close interdependence between PNX-14 and renal function (Supplementary Table S1). Spearman correlation between PNX-14 and ordinal CKD stage confirmed a strong positive monotonic trend (*ρ*=0.536, *p* < 0.001; Supplementary Table S2). Effect sizes for pairwise stage comparisons ranged from small (Stage 4 vs. Stage 5, Cohen's d = 0.445) to large (Stage 3 vs. HD, d = 1.107) (Supplementary Table S3).

### 3.3 Diagnostic performance of PNX-14

**Figure 1:**
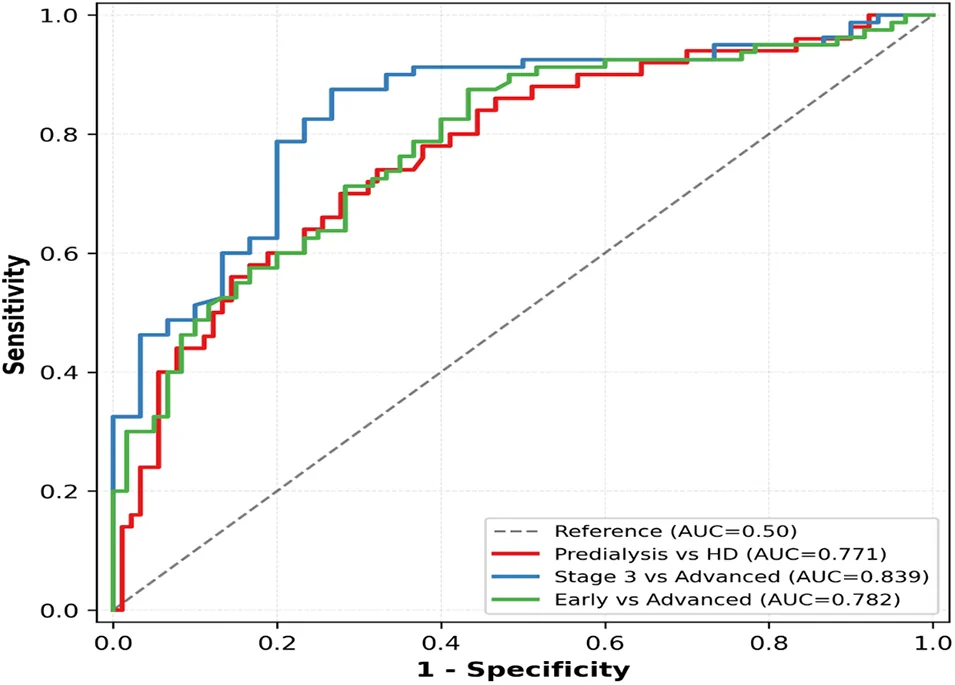
ROC curve analysis for discriminating CKD stages using Serum PNX-14. ROC curves showing diagnostic performance of serum PNX-14 in distinguishing CKD stages. The highest AUC (0.839) was observed for Stage 3 vs. advanced disease (Stage 5 + HD). AUC, area under the curve; HD, hemodialysis.

ROC curve analysis was performed to evaluate the ability of serum PNX-14 to discriminate between different stages of CKD (Table 4, Figure 1). The highest diagnostic performance was observed in distinguishing early-stage disease (Stage 3) from advanced-stage disease (Stage 5 + HD) [AUC: 0.839 (95% CI: 0.755-0.913)]. For this comparison, the optimal cut-off value was determined to be 19.59 pg/mL (87.5% sensitivity, 73.3% specificity). In distinguishing pre-dialysis patients (Stage 3 + 4 + 5) from HD patients, the AUC was 0.771, with a cut-off value of 27.39 pg/mL (70.0% sensitivity, 72.2% specificity).

**Table 4:** Diagnostic performance of serum PNX-14 in discriminating CKD stages.

Comparison	AUC (95% CI)	Cut-off (pg/mL)	Sensitivity (%)	Specificity (%)
Pre-dialysis vs. HD	0.771 (0.689–0.849)	27.39	70.0	72.2
Stage 3 vs. Advanced (5 + HD)	0.839 (0.755–0.913)	19.59	87.5	73.3
Early (3 + 4) vs. Advanced (5 + HD)	0.782 (0.698–0.853)	19.59	87.5	56.7

ROC curve analysis was performed to evaluate the discriminative ability of serum PNX-14. AUC, area under the curve; CI, confidence interval; HD, hemodialysis. Cut-off values were determined using Youden's index (maximum sensitivity + specificity-1). 95% confidence intervals were calculated using bootstrap method (1,000 iterations).

### 3.4 Hemodialysis kinetics

In HD patients (*n* = 50), pre-dialysis and post-dialysis PNX-14 levels were compared (Table 5). While the pre-dialysis PNX-14 level was 67.02 ± 53.63 pg/mL, the post-dialysis level was measured as 63.56 ± 44.39 pg/mL. No statistically significant difference was observed between the two measurements (Wilcoxon signed-rank test, *p* = 0.152). An increase in post-dialysis PNX-14 levels was observed in 64% of patients (*n* = 32), whereas a decrease was found in 36% (*n* = 18). Although more patients showed an increase than a decrease, the mean change was slightly negative because the reductions were larger in magnitude than the increases. The mean change was −3.46 ± 45.15 pg/mL (18.3 ± 57.2%). No significant correlation was detected between Kt/V and pre-dialysis (r=+0.244, *p* = 0.087) or post-dialysis (r=+0.143, *p* = 0.323) PNX-14 levels.

**Table 5:** Comparison of serum PNX-14 levels before and after hemodialysis.

Parameter	Pre-Dialysis	Post-Dialysis	Change (pg/mL)	Change (%)	*P* value
PNX-14 Levels					
Mean ± SD	67.02 ± 53.63	63.56 ± 44.39	-3.46 ± 45.15	18.3 ± 57.2	0.152
Median (IQR)	43.14 (23.78–95.71)	45.81 (33.70–84.63)	4.90	16.4	-
Kt/V Correlation					
Correlation (r)	+0.244	+0.143	-	-	0.087/0.323

*n* = 50 HD patients. Paired comparison: Wilcoxon signed-rank test. Correlation: Spearman analysis (*p*-values: pre/post).

### 3.5 Correlation of PNX-14 with clinical parameters

In Spearman correlation analysis, serum PNX-14 levels showed strong correlations with kidney function parameters (Table 6). PNX-14 was positively correlated with creatinine and PTH, and negatively correlated with eGFR (all *p* < 0.001). In the subgroup analysis performed among pre-dialysis patients (Stages 3, 4, and 5), PNX-14 retained its significant correlations with creatinine, eGFR, urea, and PTH; however, its correlations with phosphorus, calcium, hemoglobin, and albumin were no longer statistically significant.

**Table 6:** Correlation between serum PNX-14 and clinical parameters.

Parameter	All Patients (*n* = 140)	Pre-dialysis Only (*n* = 90)
Creatinine (mg/dL)	+0.512 (<0.001)***	+0.410 (<0.001)***
eGFR (mL/min/1.73 m^2^)	−0.507 (<0.001)***	−0.369 (<0.001)***
BUN (mg/dL)	+0.367 (<0.001)***	+0.320 (0.002)**
PTH (pg/mL)	+0.438 (<0.001)***	+0.368 (<0.001)***
Phosphorus (mg/dL)	+0.313 (<0.001)***	+0.157 (0.138)
Calcium (mg/dL)	−0.339 (<0.001)***	−0.197 (0.062)
Hemoglobin (g/dL)	−0.242 (0.004)**	−0.177 (0.095)
Serum Albumin (g/dL)	−0.188 (0.026)*	−0.150 (0.158)
Spot Urine ACR (mg/g)	-	+0.327 (0.002)**

Spearman correlation analysis. BUN: Blood urea nitrogen; PTH: Parathyroid hormone; eGFR: Estimated glomerular filtration rate; ACR: Albumin-to-creatinine ratio.

Pre-dialysis group includes CKD Stage 3, 4, and 5 patients (HD patients excluded). ACR data available only for pre-dialysis patients.

* *p* < 0.05.

** *p* < 0.01.

*** *p* < 0.001.

### 3.6 Multivariable analysis and *post hoc* power

To determine whether serum PNX-14 was associated with renal function independently of demographic factors, multivariable linear regression was performed using log-transformed PNX-14 as the dependent variable. In the whole cohort, eGFR remained independently associated with PNX-14 after adjustment for age, sex, BMI and PTH (*β*=−0.018 per mL/min/1.73 m^2^, 95% CI −0.028 to −0.009, *p* < 0.001; standardized *β*=−0.35), as did PTH (*β*=0.0003 per pg/mL, 95% CI 0.0000–0.0006, *p* = 0.048), whereas age, sex and BMI were not (all *p* > 0.20). The model explained a modest proportion of the variance in PNX-14 (R^2^ = 0.231, adjusted R^2^ = 0.202), indicating that most of the variability in circulating PNX-14 is not accounted for by conventional renal and demographic parameters. Variance inflation factors were below 3 for all covariates. In the pre-dialysis subgroup (*n* = 90), PTH remained independently associated with PNX-14 (*β*=0.0015, *p* = 0.006), whereas eGFR did not reach significance after adjustment (*p* = 0.458), and the albumin-to-creatinine ratio was not independently associated (*p* = 0.981).

In multivariable binary logistic regression comparing Stage 3 with advanced disease (Stage 5 + HD), each 10 pg/mL increment in serum PNX-14 was independently associated with advanced disease after adjustment for age, sex and BMI (OR = 2.10, 95% CI 1.16–3.79, *p* = 0.014). Consistent with the ordinal models presented in Supplementary Table S1, this association was attenuated to the null when creatinine and eGFR were added to the model (OR = 1.02, 95% CI 0.88–1.18, *p* = 0.80), reflecting the collinearity between PNX-14 and the measures that define CKD staging.

A *post hoc* power analysis was performed for the primary comparison. The observed between-group effect for log-transformed PNX-14 across the four groups was large (*η*^2^ = 0.248; Cohen's f = 0.574), corresponding to a statistical power exceeding 99% at *α*=0.05 with the available sample of 140 patients; the smallest effect detectable with 80% power under the same conditions was f = 0.29. For the paired comparison of pre- and post-dialysis PNX-14 (*n* = 50), the SD of the within-patient change was 45.1 pg/mL, so the study had 80% power to detect a mean change of 17.9 pg/mL, approximately 27% of the mean pre-dialysis concentration. Differences smaller than this threshold cannot be excluded.

A sensitivity analysis using Dunn's test with Bonferroni adjustment in place of the Mann–Whitney U test with Holm-Bonferroni correction confirmed the significance of the Stage 3 vs. Stage 5, Stage 3 vs. HD, and Stage 4 vs. HD comparisons (all adjusted *p* < 0.005), whereas the Stage 5 vs. HD comparison was no longer significant under this more conservative procedure (adjusted *p* = 0.209). This comparison should therefore be interpreted with caution.

### 3.7 Serum PNX-14 according to sex

Serum PNX-14 did not differ between men and women in the cohort as a whole (median 23.60 pg/mL, IQR 18.53–39.05 in men vs. 23.49 pg/mL, IQR 18.69–49.40 in women; *p* = 0.943). No difference was observed in the predialysis group (21.46, IQR 16.48–30.86 vs. 20.22, IQR 17.79–30.07; *p* = 0.841) or in the HD group (37.25, IQR 22.25–100.03 vs. 58.80, IQR 34.58–84.97; *p* = 0.320), nor within any individual CKD stage (Stage 3, *p* = 0.404; Stage 4, *p* = 0.271; Stage 5, *p* = 0.389), although the stage-specific comparisons were limited by small numbers, particularly in Stage 3, which included only five women. In HD patients, postdialysis PNX-14 also did not differ by sex (*p* = 0.133). In a two-way analysis of variance on log-transformed PNX-14, the effect of CKD group remained significant (F = 14.68, *p* < 0.001), whereas neither sex (F = 0.03, *p* = 0.867) nor the group-by-sex interaction (F = 0.55, *p* = 0.649) was significant, indicating that the stage-dependent increase in PNX-14 was of similar magnitude in men and women. Consistent with this, sex was not independently associated with PNX-14 in the multivariable model (*β*=0.128, 95% CI −0.156–0.411; *p* = 0.375).

## 4 Discussion

To the best of our knowledge, this is the first study to investigate PNX-14 levels in patients with CKD. The most important finding of our study is that serum PNX-14 levels increased significantly as kidney function deteriorated and were particularly elevated in patients requiring HD. The strong association between PNX-14 and CKD stage, together with the markedly higher levels observed in the HD group, suggests that this peptide may serve as a potential biomarker reflecting the severity of renal function loss.

There are only a limited number of clinical studies in the literature examining the role of PNX-14 in different metabolic and cardiovascular diseases. In a study conducted by Akdu et al. in hypertensive patients, serum PNX-14 and PNX-20 levels were found to be significantly lower in hypertensive patients compared with the control group (15). Similarly, Can et al. reported that PNX-14 and PNX-20 levels were significantly lower in patients with type 2 diabetes mellitus (T2DM) than in healthy controls, were negatively correlated with BMI, fasting blood glucose, HbA1c, and insulin resistance, and showed good diagnostic performance for the diagnosis of T2DM (16). At first glance, the finding in these studies that PNX-14 levels are reduced in metabolic disorders appears to conflict with our observation that PNX-14 levels increase as CKD stages progress. However, this difference may be explained by several possible interpretations. For example, in studies attributing low PNX-14 levels to diabetes and hypertension, reduced PNX-14 was considered a risk factor contributing to disease development, whereas the elevated levels observed during the course of CKD may suggest a compensatory protective response to progressive renal injury. Such compensatory peptide responses are a well-defined mechanism in chronic diseases. For instance, the progressive increase in brain natriuretic peptide (BNP) levels in heart failure reflects an endogenous cardioprotective response aimed at protecting the myocardium and maintaining hemodynamic balance (17). Likewise, the increase in erythropoietin (EPO) levels in chronic hypoxia represents a physiological compensatory mechanism intended to improve tissue oxygenation (18). Indeed, the renoprotective properties of PNX-14 demonstrated experimentally by Öz et al. have shown that this peptide protects renal tissue against oxidative stress and injury (13). In this context, the increase in PNX-14 levels with CKD progression may be interpreted, much like BNP and EPO, as an endogenous defense mechanism developed by the body against ongoing kidney damage.

Alternatively, the increase in PNX-14 levels with advancing CKD stage observed in our study may also be related to other mechanisms. First, in renal failure, reduced renal clearance leads to increased plasma levels of many peptides and proteins (19). PNX-14 is a small peptide consisting of 14 amino acids with a molecular weight of 1.6 kDa (8), and it may plausibly be cleared by the kidney through glomerular filtration and tubular metabolism. However, as eGFR progressively declines, PNX-14 clearance may also decrease, resulting in its accumulation in the circulation. Second, SMIM20, the precursor protein of PNX-14, is known to play a critical role in mitochondrial energy production (11), and mitochondrial dysfunction caused by oxidative stress, chronic inflammation, and the accumulation of uremic toxins (e.g., indoxyl sulfate and *p*-cresyl sulfate) is clearly an important mechanism contributing to the progressive deterioration of kidney function in CKD patients (20). Therefore, considering that PNX-14 may be involved in cellular energy metabolism and the adaptive response to oxidative stress (21), it is plausible to hypothesize that PNX-14 levels may increase as an adaptive response to the growing mitochondrial stress associated with CKD progression. However, prospective studies investigating the renal elimination of PNX-14, its levels in different kidney diseases, and its relationship with clinical outcomes are needed to confirm this hypothesis. Three mechanistic considerations deserve emphasis. First, with respect to renal handling, PNX-14 is well below the glomerular filtration threshold of approximately 60 kDa, so free peptide would be expected to be filtered and largely catabolised by proximal tubular cells, as occurs with other low-molecular-weight peptides. Progressive nephron loss would reduce both filtered load and tubular catabolic capacity, and impaired tubular metabolism may be quantitatively more important than reduced filtration for peptides of this size. Second, with respect to the receptor pathway, PNX-14 signals through GPR173, a G protein-coupled receptor that acts through cyclic AMP-dependent pathways and has been linked to attenuation of oxidative stress and inflammatory signalling; whether GPR173 is expressed in human kidney tissue, and whether receptor expression changes with CKD progression, has not been established, and this remains a substantial gap in the mechanistic argument. Third, with respect to the mitochondrial axis, the shared precursor of PNX-14, SMIM20, participates in the assembly of cytochrome c oxidase, and proximal tubular cells are among the most mitochondria-rich in the body and are particularly vulnerable to the bioenergetic failure that accompanies uraemia. Increased transcription of SMIM20 under such conditions could raise circulating PNX-14 as a by-product of an adaptive mitochondrial response rather than through any kidney-specific mechanism. It should be emphasized that all three pathways are hypothetical in the present context: the current study measured only circulating peptide concentrations and provides no direct evidence of renal handling, receptor expression or mitochondrial activity. Tissue expression studies, clearance measurements using labelled peptide, and experiments in models of chronic rather than acute kidney injury would be required to distinguish between them.

In our study, the comparison of pre-dialysis and post-dialysis PNX-14 levels in HD patients provides important insights into the dialysis kinetics of this peptide. The absence of a statistically significant difference between pre-dialysis and post-dialysis PNX-14 levels suggests that PNX-14 is not effectively removed by HD membranes. In addition, no significant correlation was demonstrated between Kt/V, an indicator of dialysis adequacy, and PNX-14 levels. These findings may be explained by several possible mechanisms. Assuming that the protein-binding properties of PNX-14 are not yet known, as noted above, although PNX-14 is a small peptide, a high protein-binding capacity could plausibly limit its passage through the dialysis membrane. Indeed, Horio et al. showed that similar peptides (ANP and BNP) exhibit different kinetic behaviors during HD and that these differences are related to their protein-binding properties (22). A second possibility may be related to the continuous release of PNX-14 from tissue reservoirs or ongoing endogenous production. Mathematical models have demonstrated that protein-bound molecules may show a post-dialytic rebound due to redistribution from tissue compartments into plasma after HD (23). This phenomenon may have compensated for any limited clearance occurring during dialysis and thereby contributed to the maintenance of serum PNX-14 levels. Finally, the dialysis modality used may also have contributed to this result. It has been shown that some peptide-structured molecules are inadequately cleared by conventional HD, whereas their clearance can be achieved with advanced dialysis techniques (24). If PNX-14 shares similar protein-binding characteristics, it may not have passed effectively through standard HD membranes. In addition, because the type of dialysis membrane used in our study (high-flux vs. low-flux) was not recorded, the effect of membrane permeability on PNX-14 kinetics could not be analyzed. Since there are no previous studies in the literature investigating the dialysis kinetics of PNX-14, our findings provide the first data in this field and constitute an important starting point for future research. Two further considerations qualify this interpretation. Ultrafiltration removes plasma water during a dialysis session and concentrates non-diffusible solutes, so an unchanged post-dialysis concentration is compatible with a modest absolute reduction in total peptide mass being offset by hemoconcentration; because ultrafiltration volumes were not recorded, this could not be quantified, and the finding should be described as an absence of a detectable fall in concentration rather than as proven non-clearance. In addition, the study was powered to detect a change of approximately 18 pg/mL in this subgroup, so smaller but potentially meaningful reductions could have escaped detection. It is also relevant that urea and creatinine fell substantially over the same sessions, with mean reduction ratios of 68.1% and 63.1% respectively, which confirms that dialysis was adequate and that the stability of PNX-14 is unlikely to be explained by inadequate treatment.

In the subgroup analysis performed among pre-dialysis patients (Stages 3, 4, and 5; *n* = 90), PNX-14 maintained its significant correlations with creatinine, eGFR, urea, and PTH, whereas its correlations with phosphorus, calcium, hemoglobin, and albumin became non-significant. With the progression of CKD, major complications such as CKD-related mineral and bone disorder (CKD-MBD), anemia, and malnutrition develop (25). Previous studies have shown that PTH levels begin to rise in the early stages of the disease, whereas abnormalities in calcium and phosphorus levels (particularly when GFR falls below 40 mL/min/1.73 m^2^), as well as decreases in hemoglobin and albumin, become more apparent only in advanced stages (26). The finding that PNX-14 correlated with PTH in pre-dialysis patients (r=+0.368, *p* < 0.001), but not with parameters of mineral metabolism, anemia, or malnutrition, suggests that PNX-14 may change in parallel with PTH at a stage when these late complications have not yet become clearly established. Indeed, in the analysis of the entire cohort, which included the HD group in whom mineral disturbances, anemia, and malnutrition were also more pronounced, PNX-14 retained significant associations with all these parameters. These findings suggest that PNX-14 may have characteristics of a kidney-specific biomarker and may reflect loss of renal function independently of the general metabolic complications associated with CKD. This interpretation must be qualified, however, because treatments directed at CKD-MBD were not recorded and act directly on PTH concentrations; the PNX-14–PTH association described here may therefore be attenuated or exaggerated by treatment status in a direction that cannot be determined from these data.

In our study, ROC curve analysis was performed to assess the ability of PNX-14 to discriminate between different stages of CKD, and PNX-14 demonstrated good diagnostic performance in distinguishing early-stage disease (Stage 3) from advanced-stage disease (Stage 5 + HD), with an AUC of 0.839 (27). This level of performance is comparable to that reported for other candidate biomarkers in the recent literature. Indeed, Chai et al. showed that NGAL (AUC: 0.841), hs-cTnT (AUC: 0.882), and LOX-1 (AUC: 0.859) also exhibited similar diagnostic accuracy (28).

Sex distribution differed significantly across the study groups, which raises the possibility that the stage-dependent gradient in PNX-14 reflected changing sex composition rather than renal function. Our analyses do not support this. PNX-14 was closely similar in men and women overall, within the predialysis and HD groups and within each individual CKD stage, sex was not independently associated with PNX-14 after adjustment, and the group-by-sex interaction was not significant, indicating that the increase across stages was of comparable magnitude in both sexes. This appears to be the first direct comparison of circulating PNX-14 between adult men and women: published human studies have been confined to single-sex populations, including women with polycystic ovary syndrome, girls with precocious puberty and men with obesity, so no reference for a sex difference in adults previously existed. Given that phoenixin was originally characterized as a reproductive peptide acting through GPR173 on gonadotropin-releasing hormone neurons, the absence of a detectable difference is worth noting, although our stage-specific comparisons were underpowered — Stage 3 included only five women — and gonadal status was not recorded, so a modest sex effect cannot be excluded.

A further question is how the concentrations observed here compare with those reported in individuals without kidney disease. Direct comparison across published cohorts is constrained by the absence of assay standardization: reported serum PNX-14 concentrations in control populations differ by more than an order of magnitude between commercial kits, and the diagnostic thresholds derived from them differ accordingly (16). The most informative comparison available is a cross-sectional study of adults with T2DM and preserved renal function, conducted at the same center and using the same commercial ELISA kit, in which mean PNX-14 rose from 30.7 ± 11.4 pg/mL in normal-weight participants to 95.4 ± 38.2 pg/mL in those with morbid obesity, with a strong positive correlation with body mass index (29). Against that reference, the concentrations in our predialysis groups were not high in absolute terms: Stage 3 patients, whose mean BMI was 30.7 kg/m^2^, had a mean PNX-14 of 19.4 pg/mL, whereas participants of comparable adiposity without CKD had values approximately threefold higher. The HD group, despite having the lowest mean BMI in our cohort, had concentrations roughly twice those of normal-weight participants in that study. These comparisons are informal and are confounded by differences in diabetes status, adiposity, comorbidity and analytical run, and they cannot substitute for a contemporaneously assayed control group. They indicate that the stage-dependent gradient reported here should be interpreted as a within-cohort relationship rather than as evidence that PNX-14 is elevated in CKD relative to the general population. It is also notable that BMI was not independently associated with PNX-14 in our multivariable models, which may reflect the restricted range of adiposity in this cohort or the dominant influence of renal function on circulating concentrations.

A related question is whether pharmacological treatment could itself have shaped the concentrations reported here. We are not aware of any study examining the effect of any drug class on circulating PNX-14, so the question can at present be addressed only mechanistically. In predialysis CKD the most relevant agents are those directed at mineral and bone disorder, which lower parathyroid hormone — the variable most strongly associated with PNX-14 after creatinine and eGFR — although variation in treatment intensity would be expected to attenuate that association rather than to create it; erythropoiesis-stimulating agents, which relieve the tissue hypoxia to which the SMIM20–mitochondrial axis has been linked; and treatments that alter body weight or metabolic state, given the reported positive association of PNX-14 with adiposity and its lower concentrations in T2DM and hypertension (15, 16, 29). In the HD group one pharmacological influence is specific and unavoidable: predialysis samples were drawn before anticoagulation, whereas postdialysis samples followed a full session of heparin exposure. Heparin releases lipoprotein lipase and generates free fatty acids that compete for albumin binding sites, which raises the measured free concentration of protein-bound solutes during hemodialysis, largely as an *in vitro* artifact dependent on continuing lipase activity in the sample tube (30). Since we have argued that protein binding may explain the apparent non-clearance of PNX-14, heparin-driven displacement could in principle raise the measured postdialysis value and mask a genuine intradialytic fall. Future studies should record medication use prospectively and, for intradialytic sampling, use heparin-free or citrate anticoagulation or add a lipase inhibitor at the time of collection.

Several limitations should be considered when interpreting these findings. First, the cross-sectional design permits only the description of associations at a single time point and does not allow temporal or causal inferences; whether serum PNX-14 rises before, alongside, or as a consequence of declining renal function cannot be determined from these data. Second, this was a single-center study conducted in one geographical region, and the generalizability of the results to other populations, ethnicities and dialysis practices remains to be established. Third, although the total sample of 140 patients provided ample power for the primary between-stage comparison, the individual groups were modest in size, and the study was not powered to detect the smaller differences between adjacent stages; the non-significant Stage 3 vs. Stage 4 and Stage 4 vs. Stage 5 comparisons should therefore be regarded as inconclusive rather than as evidence of absence. Fourth, comorbidity status and concomitant medication use were not systematically recorded, so conditions such as diabetes mellitus, hypertension and cardiovascular disease, and treatments such as renin-angiotensin system blockers, vitamin D analogues, phosphate binders and erythropoiesis-stimulating agents, could not be included as covariates; residual confounding by these factors cannot be excluded. This limitation deserves particular emphasis in relation to mineral metabolism and anemia: vitamin D analogues, calcimimetics and phosphate binders directly modify parathyroid hormone concentrations, and erythropoiesis-stimulating agents directly modify hemoglobin, so the associations reported here between PNX-14 and these variables may partly reflect treatment rather than disease severity. We are not aware of published data on the effect of any of these drug classes on circulating PNX-14, and the direction and magnitude of such a bias therefore cannot be estimated. Prospective recording of comorbidity and medication should be regarded as a minimum requirement for future studies of this peptide in CKD. Fifth, no healthy control group was included and no reference samples were assayed alongside the study samples, so the absolute concentrations reported here cannot be positioned against values expected in individuals with preserved renal function. This limitation is compounded by the absence of an internationally standardized PNX-14 assay: control values reported in the literature vary by more than an order of magnitude between commercial kits, so published control ranges cannot be borrowed as a substitute. The extent to which PNX-14 concentrations in Stage 3 CKD differ from those in individuals with preserved renal function therefore remains unknown, and the stage-dependent gradient described here is interpretable only as a within-cohort comparison. Sixth, although both low-flux and high-flux dialysers were in routine use, the membrane type was not recorded at the patient level, and dialysis vintage, ultrafiltration volume and residual renal function were likewise unavailable, so their influence on intradialytic PNX-14 behaviour could not be examined. Seventh, PNX-14 was measured with a single commercial ELISA kit without an independent confirmatory method such as mass spectrometry, and no internationally standardized assay or reference interval for this peptide currently exists, which limits comparability with other laboratories. Serum creatinine was measured by the Jaffe method, which is more susceptible to interference from non-creatinine chromogens than enzymatic assays; although this is the assay in routine clinical use at our center and is subject to internal and external quality control, some misclassification of eGFR-based CKD stage cannot be excluded. Finally, no longitudinal follow-up was undertaken, so the relationship between PNX-14 and clinically relevant outcomes such as progression to kidney replacement therapy, cardiovascular events or mortality remains unknown.

In conclusion, this study demonstrated that serum PNX-14 levels increase progressively with advancing CKD stage and show strong correlations with renal function parameters. The absence of a significant fall in PNX-14 across a dialysis session, together with its associations with standard renal biomarkers and PTH, indicates that the peptide is not readily cleared by conventional HD and that its concentrations track the severity of renal impairment. These observations are hypothesis-generating rather than confirmatory. Because the study was cross-sectional, no causal or temporal relationship between PNX-14 and CKD progression can be inferred, and the discriminative performance reported here was obtained in the same cohort in which the cut-off was derived, without independent or external validation. The proposed cut-off value should therefore be regarded as provisional and is not yet suitable for clinical decision-making. PNX-14 should at present be viewed as an exploratory candidate marker whose incremental value over eGFR and creatinine remains to be demonstrated. In light of these findings, we believe that prospective longitudinal cohort studies, assay standardization, validation studies across different kidney diseases, and investigations of its ability to predict disease progression are needed to evaluate the clinical utility of PNX-14 in CKD management.

## Data Availability

The original contributions presented in the study are included in the article/Supplementary Material, further inquiries can be directed to the corresponding author.
