# Supplementary material for: Serum phoenixin-14 levels in chronic kidney disease and hemodialysis patients: a cross-sectional study
Source: Front Med (Lausanne). 2026 Sep 7;13:1896542. doi: 10.3389/fmed.2026.1896542 (PMC13590326; doi:10.3389/fmed.2026.1896542)
Supplement: Supplementary file 1 [file Table1.docx]

**Supplementary Material**

Ordinal logistic regression demonstrated a dose-response relationship: each 10 pg/mL increase in PHX14 was associated with 1.25-fold higher odds of advanced CKD stage (OR=1.247, 95% CI: 1.122–1.385, p<0.001) after adjusting for age, sex, and BMI (Table S1). Spearman correlation confirmed strong monotonic trend (ρ=0.536, p<0.001; Table S2). Effect sizes ranged from small (Stage 4 vs 5, Cohen’s d=0.445) to large (Stage 3 vs HD, d=1.107), confirming clinically meaningful differences between groups (Table S3).

**Table S1. Ordinal Logistic Regression Analysis: Association Between Serum PHX14 Levels and CKD Stage Progression**

| **Model** | **Adjustment Variables** | **OR (per 10 pg/mL)** | **95% CI** |
| --- | --- | --- | --- |
| Model 1 | Unadjusted | 1.295*** | 1.158–1.448 |
| Model 2 | + Age, Sex, BMI | 1.247*** | 1.122–1.385 |
| Model 3 | + Creatinine, eGFR | 1.022 | 0.878–1.189 |
| Model 4 | + PTH, Hemoglobin | 1.020 | 0.876–1.186 |

*Ordinal logistic regression with CKD stage (3 < 4 < 5 < HD) as the ordinal outcome. OR: Odds ratio for advancing one stage per 10 pg/mL increase in PHX14. Model 1: Unadjusted. Model 2: Adjusted for age, sex, and body mass index (BMI). Model 3: Additionally adjusted for creatinine and estimated glomerular filtration rate (eGFR). Model 4: Additionally adjusted for parathyroid hormone (PTH) and hemoglobin. *p<0.05, **p<0.01, ***p<0.001. n=140.*

**Table S2. Spearman Correlation Analysis: PHX14 and CKD Stage as Ordinal Variable**

| **Analysis** | **Spearman's ρ** | **P-value** |
| --- | --- | --- |
| PHX14 vs CKD Stage (ordinal: Stage 3=1, Stage 4=2, Stage 5=3, HD=4) | 0.5358 | 0.00000000*** |

*Spearman rank correlation between serum PHX14 levels and CKD stage treated as an ordinal variable (Stage 3=1, Stage 4=2, Stage 5=3, Hemodialysis=4). ρ = 0.5358 indicates strong positive monotonic correlation. ***p<0.001. n=140.*

**Table S3. Pairwise Comparisons Between CKD Stages with Effect Sizes**

| **Comparison** | **Median Δ (pg/mL)** | **Cohen's d** | **Effect Size** | **P-value** |
| --- | --- | --- | --- | --- |
| Stage 3 vs Stage 4 | +4.07 | 0.478 | Small | 0.043575* |
| Stage 3 vs Stage 5 | +5.77 | 0.691 | Medium | 0.000154*** |
| Stage 3 vs HD | +25.26 | 1.107 | Large | 0.000000*** |
| Stage 4 vs Stage 5 | +1.70 | 0.445 | Small | 0.071274 |
| Stage 4 vs HD | +21.19 | 0.912 | Large | 0.000031*** |
| Stage 5 vs HD | +19.49 | 0.482 | Small | 0.016616* |

*Pairwise comparisons using Mann-Whitney U test. Cohen's d: standardized mean difference (effect size). Effect size interpretation: <0.2 = negligible, 0.2–0.5 = small, 0.5–0.8 = medium, >0.8 = large. *p<0.05, **p<0.01, ***p<0.001. HD: Hemodialysis. n=140. P-values shown here are unadjusted (nominal); the multiple-comparison (Holm-Bonferroni) correction and corresponding significance decisions are reported in the main manuscript (Table 2b). Accordingly, the Stage 3 vs Stage 4 and Stage 4 vs Stage 5 comparisons are not statistically significant after correction.*

*Methodological Note: These analyses complement the main manuscript by providing ordinal and effect size perspectives on PHX14-stage associations. The ordinal logistic regression is methodologically preferable to linear regression for ordered categorical outcomes. The attenuation of PHX14's association after adjustment for kidney function parameters (Models 3-4) reflects the biological relationship where PHX14 elevation is intrinsically linked to declining renal function, which itself defines CKD staging. This pattern is expected and does not diminish the clinical utility of PHX14 as a biomarker.*
